# Empowered by participation: Patients’ and caregivers’ experiences in the law clinic. Multidisciplinary dementia research at the intersection of law and medicine

**DOI:** 10.1177/13872877261487378

**Published:** 2026-09-15

**Authors:** Henna Nikumaa, Kaisa Näkki, Jenni Niinimäki, Mervi Issakainen, Elena Ppali, Juho Kalapudas, Noora Suhonen, Kaijus Ervasti, Anna Mäki-Petäjä-Leinonen, Eino Solje

**Affiliations:** 1 Law School, 163043 University of Eastern Finland, Joensuu, Finland; 2 Institute of 220881 Clinical Medicine – Neurology, University of Eastern Finland, Kuopio, Finland; 3 A.I. Virtanen Institute for Molecular Sciences, University of Eastern Finland, Kuopio, Finland; 4 Neuro Center – Neurology, 60650 Kuopio University Hospital, Kuopio, Finland; 5 Neurocenter, Neurology, Oulu University Hospital, Oulu, Finland; 6 Research Unit of Clinical Medicine, Neurology, 6370 University of Oulu, Oulu, Finland

**Keywords:** Alzheimer's disease, dementia, empowerment, feasibility study, law clinic, mixed-methods

## Abstract

**Background:**

A pilot study, the Law Clinic, implemented extensive cognitive, neuropsychiatric, and legal assessments within an interdisciplinary framework. A study design of this kind has not been previously applied. The study was necessary to generate knowledge on the relationship between symptoms of dementia and patients’ legal status.

**Objective:**

The pilot aimed to determine, through participants’ experiences, whether expanding such multi-component research is ethically sustainable, and whether clinical studies that combine medical and legal perspectives of this nature can feasibly be conducted.

**Methods:**

20 people with Alzheimer's disease and related disorders and 20 caregivers (spouses) participated in the study, which included several assessments and interviews for both parties. In the final interview, participants reflected on their experiences participating in the study. The transcribed material was analyzed using inductive content analysis to identify key themes related to participation and perceived burden.

**Results:**

Participants reported positive experiences, with 90% indicating no burden. Most described the study as meaningful, empowering, and even enjoyable. Separate interviews for patients and caregivers were considered essential for open communication and comprehensive perspectives. The findings support scaling up the Law Clinic Study.

**Conclusions:**

The novel Law Clinic Study protocol proved sustainable and feasible. Thoughtful design, and multidisciplinary expertise can ensure respectful and meaningful engagement in research for people with Alzheimer's disease and related disorders and their caregivers. Our findings suggest that research addressing complex and sensitive issues through broad interdisciplinary methods can be ethically sound, generate unique knowledge that would otherwise remain inaccessible, and may even prove empowering for participants.

## Introduction

Research on people with Alzheimer's disease and related disorders (ADRD) and their family members spans across multiple academic disciplines. However, research largely ignored the perspectives of people with ADRD until the 1990s.^1^ This exclusion from research has been reinforced by prevailing assumptions that people with ADRD are incompetent and incapable of reflecting on, or articulating their views.^2–4^ These perceptions have contributed to their marginalization in research contexts. Additionally, the dominant biomedical focus on neuropathology has often limited attention to the lived experiences, abilities and social interactions of people with ADRD.^5,6^ Perspectives of people with ADRD in legal research remain particularly rare.^7,8^

Understanding the legal dimensions of dementia, such as vulnerability to mistreatment, risk for criminal and socially inappropriate behavior, changes related to legal capacity, and rights protection, requires a foundation of medical insight. Without clinical knowledge of disease stage, existing setting of symptoms and signs and cognitive domains affected, legal assessments risk oversimplification or misrepresentation. For example, a person with ADRD may demonstrate marked anosognosia, firmly insisting that they manage their finances and daily affairs independently despite significant cognitive decline.^9,10^ In a legal capacity assessment, this lack of insight could be misinterpreted as a deliberate refusal to cooperate or as an indication of preserved decision-making capacity. Without knowledge that anosognosia is a clinical feature of ADRD, the assessor may overestimate the individual's ability to understand risks, appreciate consequences, or make informed choices, leading to an inaccurate conclusion about their legal capacity. From the patient's perspective, changes in capacity unfold longitudinally and affect different domains in varying ways. By combining legal and medical scientific perspectives, dementia research can generate new, meaningful insights that reflect the lived experience.^11^

Despite growing awareness, persistent prejudices still surround the involvement of people with ADRD in research, both in terms of practical implementation and ethical considerations.^12–14^ Particular concern has been raised about the potential burden and distress that participation in intensive, multi-component studies may cause.^15–17^ It is therefore essential to examine the experiences of people with ADRD and their caregivers in such research contexts. Overemphasizing vulnerability can inadvertently marginalize people with ADRD and reinforce prejudiced notions of their incapacity.^18^ While the intention may be to protect, the assumption that individuals with dementia are unable to express their experiences or opinions, and therefore cannot participate in research, further entrenches stereotypes of incompetence^4,19^ and may unnecessarily prevent the research or methodology of relevant research questions. Such stigma not only undermines their equal rights but also risks obstructing the investigation of critical scientific questions.

According to Groothuijse et al.,^20^ participation in research is both possible and beneficial; researchers should avoid assumptions about the burden of participation and refrain from presuming that people with ADRD lack the ability to contribute. Findings of prior research emphasize the importance of researcher's dementia-specific communication skills and approaches that support sensitive, person-centered engagement.^21–23^ In addition, valid concerns, such as emotional strain, fatigue, and logistical challenges, must be carefully addressed through thoughtful study design.^14^ To assess the feasibility and acceptability of demanding research protocols, pilot studies are often employed.^24,25^

This article presents a pilot study, which combined extensive legal, cognitive, and neuropsychiatric assessments for people with ADRD and their primary caregivers. The interdisciplinary study incorporated equally legal and medical data collection. This novel study can be considered ethically challenging due to the several multi-component tests and assessments involved, as well as the length of the study day, particularly from the perspective of the patients’ resources to tolerate the demands of the study. Study was necessary in order to generate knowledge on the relationship between symptoms and signs related to neurodegenerative dementia and patients’ legal status and the realization of their rights.

The study is referred to as the Law Clinic. To our knowledge, the Law Clinic is the first of its kind study design globally and provides a highly promising platform for integrating legal and biomedical insights. Importantly, the Law Clinic presented in this paper differs from other law clinics. Traditionally, law clinics operate as part of legal education in universities. In a regular law clinic, law students practice providing legal advice to real clients, who may present with any type of legal issue. The clientele can be anyone from the general population.^26,27^ In contrast, our clinic specifically focuses on people with ADRD and their caregivers. In addition, unlike other clinics, our Law Clinic Study integrates legal and medical assessments to examine how ADRD affects patients’ legal capacity and the realization of their rights.

This article examines the feasibility and acceptability of the pilot phase of the Law Clinic study, which involved people with ADRD (n = 20) and their caregivers (n = 20). We examined feasibility and acceptability through how they experienced an intensive and multi-component research visit, which included several assessments and interviews conducted on the same day. The pilot had a twofold objective: first we aimed to determine, through participants’ experiences, whether expanding such multi-component research is ethically sustainable, and whether clinical studies that combine medical and legal perspectives of this nature can feasibly be conducted. The second objective was also to identify ways to minimize the possible burden placed on individuals with dementia and their caregivers.

## Methods

### Study design

The multidisciplinary Law Clinic Study examined the relationship between symptoms and signs related to neurodegenerative dementia and patients’ legal status and the realization of their rights (see Supplemental Material 1 for the additional information about the study protocol). Furthermore, the study investigated the capacity of individuals with ADRD to manage legal affairs, such as handling financial transactions, signing contracts, granting powers of attorney, or making decisions about property and personal matters, at different stages of the disease. It also examined the practical implementation of their rights, the prevalence of experienced mistreatment, and how these experiences correlated with results from neuropsychological and neuropsychiatric assessments. The aim was to understand how neurocognitive diseases, symptoms, findings, and other factors affect the legal capacity of people with ADRD, as well as the rights and legal challenges faced by both them and their caregivers.

The participants underwent various assessments and interviews. The data combined medical, legal, neuropsychological, and neuropsychiatric information with experiential knowledge gathered from individuals with dementia and their caregivers (see Supplemental Material 1). However, in this paper we do not report or discuss the results of the overall Law Clinic Study. This paper examines how the pilot phase of the Law Clinic Study was experienced by patients and their caregivers, which was conducted as a non-randomized mixed-method feasibility study. Although the primary approach of the study was qualitative, the presentation of the results also includes quantitative descriptions.

### Inclusion criteria of the participants

The inclusion criteria for the study: 1) they were 18 years or older, 2) they had received diagnostic assessment within the North Savo Welfare region 3) they had received a diagnosis of a progressive neurocognitive disease, 4) they were still capable of providing informed consent, and 5) they had an adult family member who knew them well and was also willing to participate in the study.

The inclusion criteria for the study partner: 1) 18 years or older, 2) familiar with the patient's situation and everyday life, and 3) able to participate in the research interviews.

### Data collection and analysis

This study, assessing the experiences of the study participants in Law Clinic pilot Study, used a qualitative approach to data collection and analysis. The data was collected as part of semi-structured interviews, which mainly focused on addressing legal issues. Interviews were conducted separately with people with ADRD and their caregivers. The total number of interview questions ranged from 47 to 61 for patients and from 50 to 54 for caregivers, depending on individual circumstances (see Supplemental Material 1). Most questions were closed-ended. The data used in this paper was based on the eight open questions focusing on participants’ experiences of taking part in the study. The themes of these questions addressed the societal position of people with ADRD, experiences of inclusion, perceptions of research participation and burden, the impact of separate interviews, and opportunities for feedback and improvement (see Supplemental Material 2 for the list of the eight interview questions).

The interviews were audio-recorded. It was not possible to calculate the exact duration of the final eight questions in relation to the total length of the interviews, as the discussions were meandering. The whole interviews lasted between 19 and 70 min, with a median duration of 37 min. The recordings were transcribed verbatim, resulting in a transcript spanning 758 pages, of which 128 pages concerned the final eight questions. The material was pseudonymized. The transcription was then manually checked and revised by the research team before it was further analyzed. The processing and storage of data followed the regulations on data security and data management at the researchers’ universities and research units, according to the General Data Protection Regulation (GDPR).

The transcription was initially read in full, and the parts concerning the participants’ experiences in participating in the study were extracted. The extracted material was analyzed using qualitative inductive content analysis.^28^ However, when reporting the results, we also present a few percentages of the responses for the sake of clarity.

The coding and the whole analysis was performed manually, without the assistance of software or artificial intelligence. The content analysis proceeded through a typical four-stage process: first, the transcribed material was divided into meaning units and condensed (reduction); second, condensed units were grouped and categorized (clustering); third, the categories were conceptualized (abstraction); and finally, they were reassembled in a new way so that the main categories were formed.^28^ Neuropsychological and medical data, including disease stage and disease entity, was used for contextual interpretation of the data. The data collection began in June 2023 and ended in October 2024.

To ensure researcher consensus, the analysis process and its stages were discussed among the authors. The coding decisions and categories were jointly reviewed until agreement was reached, and any differences in interpretation were resolved through iterative discussion. Trustworthiness and credibility were strengthened by involving multiple researchers in the analysis process, maintaining an audit trail of coding decisions, systematically pseudonymizing the data, and cross-checking interpretations against the original transcripts. The multidisciplinary composition of our research team further supported the transparency and reliability of the analysis, as the stages of the analysis and the interpretations were examined from several disciplinary perspectives, allowing for critical reflection and refinement throughout the process.

### Ethical approval

The Regional Medical Research Ethics Committee of Northern Savonia Hospital District approved the study protocol (2194/2022). All the participants provided written informed consent. The principles of Helsinki Declaration (2022) were rigorously followed. The study was conducted in accordance with the version of the Declaration of Helsinki that was applicable at the time ethical approval was obtained and the study was initiated. We carefully reviewed the 2024 revision and did not identify aspects that would alter the ethical conduct, analysis, or reporting of the present study. Furthermore, international ethical recommendations on consent capacity in dementia were followed. According to recommendations, the presumption should be that a person with dementia is capable of giving consent unless proven otherwise.^29^ In addition, the members of our research team have long-lasting experience working with people with ADRD, which supported careful evaluation of participants’ understanding, voluntariness, and ability to provide informed consent throughout the recruitment and interview process.

## Results

### Demographics

In the pilot phase, the study included 20 adult patients diagnosed with dementia and 20 of their primary caregivers, all of whom were spouses. All couples lived together in the same household. Fourteen couples lived in an urban area, while six couples lived in a rural area. The demographics of the study participants are presented in Table 1.

**Table 1.** Demographic data for patients and their caregivers.

Measure	Patients			Caregivers		
	Mean (Range)	% (n/N)	SD (IQR)	Mean (Range)	% (n/N)	SD (IQR)
Age (years)	70.2 (59–83)		7.6 (7.3)	70.4 (56–90)		8.4 (8.5)
Sex: % male (n/N)		65 (13/20)			45 (7/20)	
Diagnosis						
Frontotemporal dementia		15 (3/20)				
Alzheimer's disease		80 (16/20)				
Mixed dementia		5 (1/20)				
Stage of the disease						
Mild		90 (18/20)				
Moderate		10 (2/20)				
Type of living*						
Detached house		55 (11/20)				
Terraced house		15 (3/20)				
Apartment house		30 (6/20)				
Degree of education						
Comprehensive school		10 (2/20)				
Upper secondary school		45 (9/20)				
Tertiary education		45 (9/20)				

SD: standard deviation; IQR: interquartile range.

* Every couple enrolled in the study shared the same household with their partner.

All missing data were excluded using pairwise deletion.

### Thematic categories

The content analysis identified three thematic categories in the reflections of people with ADRD and their caregivers on their participation in the Law Clinic Pilot Study. The results are presented according to these three categories: (1) navigating burden, (2) ensuring dual perspectives, and (3) fostering empowerment.

We use direct quotations from the interviews both to illustrate the reporting of results and to bring the participants’ own voices into the presentation of findings. The interviewees were assigned running numbers from 1 to 20. The numbers assigned to the participants are based on the transcription order, which was not necessarily the same as the order in which the interviews were conducted. In the data excerpts, D indicates a participant with dementia, C a caregiver, and R the researcher.

### Navigating burden

Altogether 90% of the participants reported not experiencing any burden or strain at all during their participation in the study. The most typical responses to the question about burden were clear and concise, such as “*Not at all”* and “*Absolutely not – quite the opposite.”* This might not be merely an attempt to please the interviewer or a reaction rooted in authority-related fear, as participants responded to other questions in a confrontational and notably candid manner.

Some responses even reflected confusion about the question itself, as participation in the study was considered very sensible – as illustrated by the following excerpts:

D6: No worries at all, like I said, I’ve worked in healthcare myself, so I really get how this whole thing goes.

D8: Well, I don’t mind at all. It's been really pleasant. We’ve gone through things that make sense to go through.

Among the participants, 17 out of 20 people with ADRD and 19 out of 20 caregivers described the experience as positive, rewarding, and even relaxing or fun, and they valued the opportunity to contribute to meaningful work, as illustrated by the following excerpts from the data:

D2: [laughing] Nah, not at all. My stress tolerance hasn’t budged, and I haven’t gotten any more stressed.

D7: Oh, this was super chill! [laughs]

C5: I’d say it felt pretty light. Not stressful or anything like that, just easygoing overall.

Only three people with ADRD and one caregiver reflected on their participation in a way that suggested a hint of burden. Among people with ADRD, these experiences were related to pre-interview nervousness and the emotional challenge of confronting their cognitive difficulties. One participant with dementia, laughing slightly, shared that they had felt nervous about coming to the hospital and taking part in the study, explaining that every day is different for them and they never quite know what the day will bring:

D2: It was a bit nerve-wracking, yeah [laughs]. Some of the questions I’d heard before, but you know… every day is different. One day feels one way, the next another… But I guess this wasn’t super detailed like ‘how was today’ or ‘how was yesterday’…

R: Right, more about the bigger picture. Still, you came to the study – thank you for that, even if you were a bit [chuckles] nervous.

D2: Yeah, I was a little nervous, wondering what was gonna happen to us [laughs].

The emotional challenges during the neuropsychological assessment were described by two persons with ADRD. The following excerpts reveal the emotionally complex and cognitively demanding nature of participating in the study. While the participants did not report burden or overt distress, their reflections suggest that certain research tasks prompted them to confront their cognitive difficulties and evoked feelings of inadequacy or self-doubt:

D15: No, I haven’t really felt that way. But yeah, like earlier, we played that game [neuropsychological tests conducted via tablet] for a couple hours and honestly… it kinda made me feel pretty dumb.

D17: Yeah, this is… mentally kind of heavy, you know? It makes you think about stuff you haven’t really had to face before, like where you’re actually at right now. Having someone from the outside, like you, really helps dig deep and open your eyes.

Two people with ADRD also commented on the nature of cognitive tests, explaining that they had made them aware of the symptoms caused by their illness, and one even reported feeling stupid during the testing. Nevertheless, both emphasized that this did not cause them any distress or burden:

D13: Nope, not really. Those memory tests and all… yeah, that's when you notice you can’t quite recall what they asked. But it doesn’t stress me out. I’m used to it by now [laughs].

D19: Yeah, I really showed how forgetful and kinda dumb I am, like in that test where the guy read a story and I had to retell it. I was like, wow, this is intense… but yeah I don’t know.

R: So how stressful has this study felt to you?

D19: Not at all. Hasn’t bothered me one bit.

In addition to the findings reported above, one interview included a response from a person with ADRD who, when asked about burden, stated: “*It's been a tough day.”* However, it is important to note that this particular interviewee's responses were generally very brief, contained only few words, and were limited in verbal content. This person with ADRD often responded by echoing the final words of the researcher's question:

R: Has today's study felt stressful, has it been a tough day?

D16: Yeah, it's been a tough day.

The above quote may be regarded as an illustration of how dementia can increase a person's vulnerability to suggestion or persuasion, which can be linked to the study's acceptability.^30^ The quote also highlights the particular considerations involved in research interviews when the interviewee is a person with ADRD. In this case, the researcher's question was leading, and the participant responded in an accommodating manner. However, the rest of the interview content or behavior of the interviewee did not indicate any actual experience of strain or burden.

19 out of 20 caregivers reported no burden, describing the experience as light, important, and positive, as mentioned above. Some also felt that participating in the study provided a pleasant change in their daily routine: “*It's kinda nice to have a bit of change in our lives.”* However, similarly to a few persons with ADRD, a few caregivers also felt that taking part in the study brought up reflections on their current situation in relation to dementia:

C7: I wouldn’t say it was stressful, but it definitely brought some things to the surface.

R: So it gave you something to think about.

C7: Yeah, exactly.

C16: I think this has been really good in the sense that I’ve kind of come to understand the illness better myself. It's been easier to make sense of things now that I know more—even if more knowledge can be tough sometimes. Still, it helps explain what's going on around you.

Only one caregiver mentioned a situation that can be interpreted as a slight burden, related to travel costs and the effort required to persuade the person with ADRD to participate. On the other hand, they continued reflecting by saying that they try to approach participation in the study as if it were a nice day out:

C3: Financially, the trip from our city to here and back costs a bit, but… we’ve tried to treat this like a little getaway. Like a fun day trip.

In general, when asked about the possible burden of participation, most participants felt no burden at all and even reported that it was a nice break from their daily life. However, a small number of the participants, both people with ADRD and their caregivers, expressed that participation in this type of research might bring up some negative feelings since answering questions acts as a reminder of their living conditions.

### Ensuring dual perspectives

The research interviews were conducted separately for people with ADRD and their caregivers. In the discussion about the feasibility of the study, the researcher inquired about participants’ experiences regarding the separate interview arrangements. Separate interviews were considered appropriate and beneficial by both groups. Most participants responded that they understood the rationale for conducting the interviews separately very well and used words such as “*naturally*” and “*obviously”.*

People with ADRD described the approach as reasonable, understandable, and effortless. They emphasized the importance of ensuring that both individuals’ thoughts and opinions are heard. A few mentioned that they had already become accustomed to being interviewed separately from their caregiver, as illustrated by the following excerpts from the data:

D4: We’ve already done dementia research before, so I’m used to it by now. It's always the same – they interview both of us separately.

D8: It's no big deal, I’m used to it. And I’m heard just as much when it comes to Arto's health stuff.

D12: That's a really smart approach. I get it from all angles, having done research myself, even if not exactly like this. This is pretty personal in a way. But yeah, absolutely, I’m on board.

Several people with ADRD also reflected on the situation from their caregiver's perspective. They considered it important that the caregiver should have the opportunity to express their own views without the person with ADRD being present. This demonstrates capacity for emotional-cognitive reflection and mentalization, despite the presence of the disease. Some also expressed concern that their illness might place a burden on their caregiver. In addition, one person with ADRD described the importance of being interviewed separately specifically for the sake of their caregiver – so that the caregiver could address sensitive experiences more freely:

D7: Yeah, I can imagine it does add more burden on the spouse. They’ve gotta think about things from my side too and take care of all the practical stuff. But so far, it's gone just fine.

D9: She's free to share her own opinion, no matter what I’ve said. She doesn’t even know what I’ve answered. You can’t say something's wrong or doesn’t hold up, each of us has our own views, and we just try to bring them together.

D17: Honestly, it makes sense. There are things you might not want to bring up… especially if you know it's a sensitive topic for Liisa so it's better that way.

Caregivers especially viewed interviewing separately as trust-enhancing and more suitable for open communication, allowing both parties to express themselves freely and thus contributing to the overall reliability of the research findings. For some caregivers, the concern that the illness might already place a burden on them increased the importance of having a separate interview, as it provided a space to discuss sensitive issues without causing additional strain or discomfort for the person with ADRD. Caregivers more often than people with ADRD emphasized the importance of being able to share their own perspective, noting that it was easier to do so without the person with ADRD being present. They also expressed a desire to not upset or embarrass their partner or to cause an argument by raising sensitive issues:

C4: Honestly, I think this setup works really well. It just feels way more natural. I probably couldn’t talk this openly if the person with dementia was sitting right next to me – there’d be a lot I’d leave unsaid. It just feels wrong to say things like “he doesn’t do this or that.” You can be way more open when it's just me.

C11: Yeah, it's definitely better this way. Otherwise you might end up arguing – like, “that's not how it happened” [laughs]. It's just easier separately, you don’t really dare to say certain things. I mean, I wouldn’t have brought up those drunk driving stories with Harri sitting there. He gets it, though—he knows those weren’t exactly proud moments [laughs].

C14: I find it really helpful, especially since I’ve been in situations where the person with dementia is right there and we’re talking about their care, and they don’t understand everything. It feels so heavy and honestly kind of demeaning to them. And on the flip side, as a spouse, you get to speak freely. It really limits what you can say when they’re sitting next to you, you don’t want to hurt them.

Both groups, people with ADRD and their caregivers, considered separate interviewing important and emphasized on a general level the value of hearing both parties’ perspectives. Several people with ADRD specifically emphasized the importance of allowing the caregiver to express their own thoughts. In contrast, only one caregiver reflected on the importance of also hearing the views of the person with ADRD. They felt that separate interviewing helps ensure that the person with ADRD's opinions are also heard. They explained that if the interviews were conducted jointly, the person with ADRD would likely defer to their caregiver, and their own perspective might be left unspoken:

C17: Yeah, it's definitely good that it's at different times. He can say whatever he wants, and he's totally able to do that [laughs], so this setup works well. If we were here together, he’d probably let me do the talking, and his opinion wouldn’t come through. That's exactly why it has to be done like this – separately.

Overall separate interviews were considered appropriate and helpful by both people with ADRD and their caregivers. The arrangement allowed each person to speak openly, especially about sensitive topics. Caregivers felt it improved trust and research reliability, while people with ADRD often emphasized the importance of hearing their partner's voice.

### Fostering empowerment

Many participants, both patients and caregivers, viewed their involvement in the study as an example of inclusion, and their reflections echoed experiences of empowerment that the participation had brought about. The interviewees considered it important to be given opportunities to participate in research and, more broadly, in society, in ways that can be considered to strengthen their sense of agency, autonomy, and inclusion. This highlights the significance of participation not merely as a means of data collection, but as a way of recognizing and amplifying the individual's value and voice.

This emerged particularly in reflections where both people with ADRD and their caregivers expressed concerns related to the generally undesirable position of people with ADRD in society, experienced stigma, and availability of social and healthcare services. The expressed hopes reflected these concerns and conveyed a desire to strengthen the inclusion of people with ADRD and their family members both in the study and in society more broadly. The interviewees emphasized the importance of encountering people with ADRD with empathy, treating them as adults, and ensuring respectful, person-centered communication, as illustrated by the following excerpts:

D8: Of course, people shouldn’t be treated like second-class citizens. There should still be that human-to-human connection, no matter what. That kind of empathy should stick around, even with the illness, people like them, or us, should be treated with respect.

C14: In our circle, we’ve seen a lot of that. People talk past them, and even with us, someone might ask me how Aulis is doing, even though he's standing right there.

C14: They talk like he's a little kid. Their voice even changes into that baby-talk tone, and come on, they’re adults. There are definitely things that healthcare staff still need more training on, especially how to actually meet people properly. They’re adults with feelings, and yeah, communication can be tough and figuring out what they mean isn’t always easy, and they should be given time.

The above reflections on how people with ADRD are encountered highlight important considerations for research settings. Being met and treated as an equal adult was regarded as essential. Such respectful encounters were also hoped for more broadly in society, as the interviewees highlighted the stigma and negative portrayals of dementia in the media. The study was seen as a potential means of influencing public discourse by contributing more accurate information.

Both interviewed groups emphasized how important and meaningful participating in the study was for them. They considered their involvement valuable and expressed hope that the findings would benefit future individuals diagnosed with dementia and their families. The overall experience of participating in the study was described in highly positive terms. People with ADRD found it empowering, meaningful, and valuable. Caregivers echoed these sentiments, highlighting the significance and positivity of their own participation. The following excerpts illustrate the general experiences of both people with ADRD and their caregivers regarding the significance of participating in the study:

D11: Yeah, I was definitely interested in taking part. It's about my own health and well-being, so of course I want to be involved. It's kind of a challenge, but I’m up for it, especially if it helps others down the line.

C1: It's been really pleasant and nice. We’re happy to join any research that could help move things forward.

D9: Honestly, it's been amazing to have this kind of interview. These things don’t usually come up, I wouldn’t think to talk about them. But now that you’re asking, I realize life's actually pretty good, even with dementia. When I first got the diagnosis, I thought life was over. But turns out, it wasn’t. There are so many ways to keep life going strong, even with memory issues.

## Discussion

This pilot of the interdisciplinary Law Clinic Study provides evidence that participation of people with ADRD and their primary caregivers in intensive and multi-component research is feasible and does not cause excessive distress. The research day for those who participated in the Law Clinic Study involved legal, medical, neuropsychological, and experiential elements (see Supplemental Material 1). In this paper we have reported the experiences of the participation. The positive feedback from participants, both in terms of emotional experience and perceived value, supports the feasibility and acceptability of the study design. The reported burden was minimal. These findings resonate with Groothuijse et al.,^18^ who argue that researchers must be cautious not to assume participation is inherently burdensome.

This study represents a unique integration of both medical and legal dimensions within dementia research—an interdisciplinary approach that remains largely unexplored in the prior research.^7,9^ The pilot combined legal and medical components in examining feasibility, experiential quality, and emotional impact. The primarily positive feedback from participants suggests that this hybrid model not only broadens the scope of dementia research but also opens new pathways for inclusive and ethically grounded research.

Despite concerns raised in earlier literature from multiple disciplines about the potential burden of intensive research protocols on people with ADRD,^14,31^ our findings suggest that such fears may be overstated when studies are thoughtfully designed across disciplines. In this pilot, 90% of participants reported no burden, and many described the experience as enjoyable, meaningful, and even empowering. However, it is also important to acknowledge, when considering the generalizability of the results, that several factors may influence such responses: for example, social desirability or gratitude toward researchers, cognitive limitations affecting self-assessment, or the context and setting of the interview situation. In addition, the participants in the study were mostly in the mild stage of their disease and had supportive spouses, which may have influenced their positive experience of taking part in the study. This is naturally not the case for every person with ADRD, and it may limit the applicability of the findings to the broader ADRD population.

These results align with previous studies indicating that individuals with dementia are capable of engaging in reflective dialogue.^2,3,26^ However, it is important to note that in studies involving people with ADRD, it is typical for the person with ADRD to perceive various services, the quality of care received, or their overall situation more positively than their close family members do.^32^

Importantly, the study's structure, particularly the separate interviews for people with ADRD and caregivers, was viewed as appropriate and beneficial by both groups. This arrangement enhanced the reliability of the data and allowed for more candid expression of experiences, echoing findings from prior research on dyadic interviews in dementia contexts.^6,5^ Caregivers especially appreciated the opportunity to speak freely without fear of upsetting their partner, while people with ADRD recognized the importance of both of their independent voices. However, we acknowledge that conducting separate interviews with both parties may be challenging in routine clinical settings, and that differences in legal frameworks across countries may complicate the integration of legal and clinical interviews.

The reflections of participants revealed a strong desire for greater inclusion of people with ADRD in research and society.^33^ Participants also expressed hope that the study's outcomes would benefit others in similar situations. This can be considered an alignment with broader trends in patient and public involvement (PPI) literature, which advocate for the active participation of marginalized groups in shaping research agendas.^22,23,34^ Although PPI in dementia research has advanced more slowly than in other fields,^10,11,35^ our findings reinforce the view that such involvement is not only feasible but also deeply valued by participants.

While the majority of participants did not report distress, a few individuals with dementia described moments of emotional challenge, particularly during cognitive and neuropsychiatric assessments. These reflections underscore the importance of sensitive research practices. The pilot's success in minimizing burden while maintaining methodological integrity suggests that future large-scale studies can adopt similar designs with confidence.

### Strengths and limitations

One of the key strengths of this study is the richness of the data. Both people with ADRD and their caregivers considered participation meaningful, and they were not only able but also willing to reflect on their experiences. Another notable strength is the multidisciplinary nature of the research team, which included experts from the fields of medicine, neuropsychology, law, and the social sciences. The testing and interviews were conducted by researchers with expertise in working with people with ADRD. The team worked in close collaboration throughout the research process, from planning to analysis and reporting, to ensure a balanced and thorough interpretation of the interview data.

However, certain limitations should be acknowledged. First, the number of participants was relatively small. As with any feasibility study, the generalizability of the findings is limited by the sample size and the specific context in which the study was conducted. Nevertheless, 40 interviews can also be considered sufficient dataset at the pilot phase. Second, all participants were from the same region in Finland, specifically Northern Savonia, and therefore potential cultural differences should be analyzed in the future. Third, participation in a study is generally more likely among those who already consider research involvement important, which may introduce a selection bias.

In addition, we acknowledge that the phrasing and structure of some interview questions may have influenced participants’ responses and introduced elements of social desirability or acquiescence bias (see Supplemental Material 2). This is a recognized methodological challenge in qualitative interview studies, especially when working with potentially vulnerable populations. However, as described earlier, the data was rich in participants’ reflections. Furthermore, the study was conducted by a multidisciplinary team with the expertise to interpret these reflections within the broader interview context.

## Conclusion

The results of our study indicate that the Law Clinic Study design—the innovative clinical study incorporating systematic neuropsychological and neuropsychiatric assessments with legal and social interviews—is feasible and our protocol can then be adapted for a larger-scale study. In the pilot phase of the study, twenty people with ADRD and twenty caregivers participated in extensive cognitive, neuropsychiatric, and legal assessments. Their overall experience was marked by positivity – particularly feelings of empowerment and meaningful engagement. Although the study protocol was ethically untested, experiences of burden were minimal, suggesting that thoughtfully designed studies can support participation without causing additional distress. However, most of the participants were in the mild stage of their disease and each had a supportive spouse, which may have contributed to their positive participation experiences.

People with ADRD have traditionally remained underrepresented in studies focused on their rights, care, and services. Many prejudices have surrounded their involvement in research, relating to their ability to participate, to provide informed consent, and to the practical implementation of study protocols. This study demonstrates that even complex and ethically demanding questions can be addressed through bold and interdisciplinary approaches.

## Supplemental Material

sj-docx-1-alz-10.1177_13872877261487378 - Supplemental material for Empowered by participation: Patients’ and caregivers’ experiences in the law clinic. Multidisciplinary dementia research at the intersection of law and medicine Supplemental material, sj-docx-1-alz-10.1177_13872877261487378 for Empowered by participation: Patients’ and caregivers’ experiences in the law clinic. Multidisciplinary dementia research at the intersection of law and medicine by Henna Nikumaa, Kaisa Näkki, Jenni Niinimäki, Mervi Issakainen, Elena Ppali, Juho Kalapudas, Noora Suhonen, Kaijus Ervasti, Anna Mäki-Petäjä-Leinonen and Eino Solje in Journal of Alzheimer's Disease

sj-docx-2-alz-10.1177_13872877261487378 - Supplemental material for Empowered by participation: Patients’ and caregivers’ experiences in the law clinic. Multidisciplinary dementia research at the intersection of law and medicine Supplemental material, sj-docx-2-alz-10.1177_13872877261487378 for Empowered by participation: Patients’ and caregivers’ experiences in the law clinic. Multidisciplinary dementia research at the intersection of law and medicine by Henna Nikumaa, Kaisa Näkki, Jenni Niinimäki, Mervi Issakainen, Elena Ppali, Juho Kalapudas, Noora Suhonen, Kaijus Ervasti, Anna Mäki-Petäjä-Leinonen and Eino Solje in Journal of Alzheimer's Disease

## Data Availability

Upon reasonable request. Participant level data cannot be shared outside the research group due to Finnish data privacy legislation.
