# Supplementary material for: Empowered by participation: Patients’ and caregivers’ experiences in the law clinic. Multidisciplinary dementia research at the intersection of law and medicine
Source: J Alzheimers Dis. 2026 Sep 15;114(1):308–18. doi: 10.1177/13872877261487378 (PMC13675530; doi:10.1177/13872877261487378)
Supplement: Supplementary file 1 [file sj-docx-1-alz-10.1177_13872877261487378.docx]

**Supplemental Material 1**

**Empowered by participation: Patients’ and caregivers’ experiences in the Law Clinic. Multidisciplinary dementia research at the intersection of law and medicine**

**Additional information about the study protocol**

*Recruitment*

Potential participants were identified based on predefined eligibility criteria and informed about the opportunity to take part in the study. We subsequently contacted those who expressed a willingness to participate by telephone. During the initial call, they were provided with a brief overview of the research and asked for permission to send them further information. Upon receiving verbal consent, participation information sheets – both for the patient and their caregiver – were sent via email. Potential participants were given adequate time to review the material independently. A follow-up call was made a few days later to address any question and to schedule a research visit for those who expressed willingness to participate. Participation was entirely voluntary, and no attempts were made to persuade or pressure individuals into joining the study. No financial compensation was offered for participation.

*Data collection*

To begin with, the patient and their caregiver met with a research nurse at the University of Eastern Finland’s Brain Research Unit. Here the patient and the caregiver underwent an informed consent process. The research nurse informed the patient and the caregiver about the purpose and methods of the study and about their rights and ensured that participants provided written informed consent. The research nurse also collected the patient's and family member's background information. Furthermore, the research nurse administered the BDI-21 (Beck Depression Inventory) questionnaire to the patient and family member to assess for possible depressive symptoms.

After the meeting with the research nurse, the patient met with a psychologist who performed neuropsychological tests on them. These neuropsychological tests were performed without the family members’ presence and included:

- Wechsler Adult Intelligence Scale-IV (WAIS-IV) Similarities and General Comprehension subtests, to assess linguistic comprehension
- WAIS-IV Cube Tasks, to assess visual reasoning
- Wechsler Memory Scale-III (WMS-III) Immediate and Delayed Narrative Recall and Ray Auditory Verbal Learning Test (RAVLT) Word List Task, to assess linguistic memory
- Trail Making Test (TMT) and INECO Frontal Screening test (IFS), to assess executive control
- To assess numerical skills, we developed a short test with addition and subtraction tasks, a magnitude perception task, and a task assessing comprehension of numerals
- Mini Mental State Examination (MMSE), to assess orientation.

At the same time, the research nurse interviewed the caregiver without the patient's presence, mapping the patient's neuropsychiatric symptoms and functional capacity using standard and established measures used in clinical work (The Neuropsychiatric Inventory (NPI), Modified Frontal Behavioral Inventory (FBI-Mod) and the Alzheimer's Disease Cooperative Study – Activities of Daily Living Inventory (ADCS-ADL).

After these parts of the study, a legally trained researcher interviewed both the patient and the family member about the realization of rights. The interviews were conducted as structured interviews, which also included open-ended questions. The interviews were carried out separately, without the presence of the other participant.

The interview with the patient focused on questions related to:

- Management of financial and personal affairs at the time of the interview
- Legal preparedness for the future (a continuing power of attorney, an advance directive, a last will and testament).
- The participant’s own perception of their mental and legal capacity to perform various legal acts (drafting a continuing power of attorney, drafting an advance directive, making a last will and testament, deciding on the sale or donation of valuable property)
- Experiences about receiving guidance and advice
- Experiences of possible abuse or neglect before and after the onset of dementia
- Realization of the right to self-determination and autonomy before and after the onset of dementia; and
- The research interview also included a Misdemeanors and Transgressions Screener (MATS) for the patient^1^; and
- At the end of the interview, the participant was asked questions about the societal position of people living with dementia and their experience of participating in the study. For a more detailed breakdown of the questions related to participants’ experiences of taking part in the study, see Supplemental Material 2.

The interview with the caregiver was largely similar in content to the one conducted with the patient. However, the interview focuses on the caregiver’s perspective on the patient’s situation, as well as their observations of the patient. The caregiver was asked the same questions that were being asked of the patient, with the exception that the interview did not include questions about the patient’s legal preparedness for the future. In addition, the included Misdemeanors and Transgressions Screener (MATS) was a version created for caregivers.^1^

**Reference**

1. Kumfor F, Wei, G, Ries N, et al. Examining the propensity and nature of criminal risk behaviours in frontotemporal dementia syndromes and Alzheimer’s disease. *Alzheimers Dement (Amst)* 2024; 16: e12577.
