# Supplementary material for: Empowered by participation: Patients’ and caregivers’ experiences in the law clinic. Multidisciplinary dementia research at the intersection of law and medicine
Source: J Alzheimers Dis. 2026 Sep 15;114(1):308–18. doi: 10.1177/13872877261487378 (PMC13675530; doi:10.1177/13872877261487378)
Supplement: Supplementary file 2 [file sj-docx-2-alz-10.1177_13872877261487378.docx]

**Supplemental Material 2**

**Empowered by participation: Patients’ and caregivers’ experiences in the Law Clinic. Multidisciplinary dementia research at the intersection of law and medicine**

**Interview questions related to participants’ experiences of taking part in the Law Clinic pilot study**

**The last questions presented to participants with Alzheimer’s disease and related disorders at the interview:**

- In your opinion, what is the societal position of people living with dementia?
- Could you describe, for example, whether you feel able to participate and be heard, seen, and accepted as yourself?
- How could the societal position of people with dementia be improved?
- Is there anything else you would like to add regarding the topics covered in the interview?
- How would you describe your experience of participating in this research?
- How burdensome has the research been for you?
- How do you feel about the fact that you and your spouse were interviewed separately?
- Would you like to provide any additional feedback about the study?

**The last questions presented to caregivers at the interview:**

- In your opinion, what is the societal position of people living with dementia?
- Could you describe, for example, how inclusion is realized?
- How could the societal position of people with dementia be improved?
- Is there anything else you would like to add regarding the topics covered in the interview?
- How would you describe your experience of participating in this research?
- How burdensome has the research been for you?
- How do you feel about the fact that you and your spouse were interviewed separately?
- Would you like to provide any additional feedback about the study?
